# Distinct drivers of bacterial community assembly processes in riverine islands in the middle and lower reaches of the Yangtze River

**DOI:** 10.1128/spectrum.00818-24

**Published:** 2024-06-13

**Authors:** Lu Yao, Junmei Wu, Shouzhuang Liu, Hao Xing, Pei Wang, Wenjuan Gao, Zhenbin Wu, Qiaohong Zhou

**Affiliations:** 1 Key Laboratory of Lake and Watershed Science for Water Security, Institute of Hydrobiology, Chinese Academy of Sciences, Wuhan, China; 2 Australian Rivers Institute, Griffith University, Nathan, Queensland, Australia; 3 School of Environmental Studies, China University of Geosciences, Wuhan, China; Oulun Yliopisto, Oulu, Finland

**Keywords:** community assembly, stochastic, deterministic, biodiversity, river ecosystem, spatial scaling

## Abstract

Riverine islands are widespread alluvium wetlands developed in large rivers, and bacterial communities are crucial to their ecological function, yet their assembly processes are rarely addressed. The ecosystem services provided by the middle and the lower Yangtze are primarily threatened by pollution discharge from agricultural land use, and resource overutilization (e.g., embankments), respectively. Here, we assessed bacterial community assembly processes and their drivers within riverine islands in the middle Yangtze River (MR islands) and those in the lower reach (LR islands). A significant distance–decay relationship was observed, although the turnover rate was lower than that of the terrestrial ecosystem with less connectivity. Deterministic and stochastic processes jointly shaped community patterns, and the influence of stochastic increased from 26% in MR islands to 59% for those in LR islands. Meanwhile, the bacterial community in MR islands was controlled more by inorganic nitrogen availability, whereas those in LR islands were governed by pH and EC, although those factors explained a limited fraction of variation in the bacterial community. Potential indicator taxa (affiliated with *Nocardioides* and *Lysobacter*) characterized the waterway transport pollution. Overall, our study demonstrated that bacterial community dissimilarity and the importance of dispersal limitation increased concurrently along the flow direction, while distinct local factors further determined bacterial community compositions by selecting habitat-specificity taxa and particularly metabolism function. These findings enhanced our understanding of the mechanisms driving changes in bacterial communities of riverine islands subject to increased anthropogenic impacts.

**IMPORTANCE:**

Rivers are among the most threatened ecosystems globally and face multiple stressors related to human activity. However, linkages between microbial diversity patterns and assembly processes in rivers remain unclear, especially in riverine islands developed in large rivers. Our findings reveal that distinct factors result in divergent bacterial community compositions and functional profiles in the riverine islands in the middle Yangtze and those in the lower Yangtze, with substantial differentiation in deterministic and stochastic processes that jointly contribute to bacterial community assemblages. Additionally, keystone species may play important metabolic roles in coping with human-related disturbances. This study provides an improved understanding of relationships between microbial diversity patterns and ecosystem functions under environmental changes in large river ecosystems.

## INTRODUCTION

Microbial community assembly is crucial for disentangling the microbial community structure and function and subsequently ecosystem function (1). In ecology, the composition and diversity of a microbial community are impacted by the following basic ecological processes: selection, drift, speciation, and dispersal (2). Among these, niche-based deterministic processes (i.e., homogeneous selection and heterogeneous selection) emphasize that communities are structured by environmental filtering, biotic interactions, and interspecific trade-offs (2, 3). Neutral-based stochastic processes (i.e., dispersal limitation, and homogenizing dispersal) emphasize the importance of probabilistic or random dispersal, immigration, or ecological drift (4, 5). Recently, it has been generally accepted that both deterministic and stochastic processes shape microbial community assembly simultaneously (5). Thus, a null-model statistical framework has been proposed and widely used to quantify the importance of deterministic and stochastic ecological processes.

Most microbial community assembly studies have focused on soil and lake environments, while have demonstrated the major driving forces of microbial community assembly, such as mean annual temperature and precipitation, pH, soil organic matter, and soil texture (6–9). However, community structure and diversity investigations of river ecosystems are still limited, especially in riverine islands. Riverine islands are alluvium wetlands mainly formed through sediment deposition within river channels (10), which have been lately considered as hot spots of biogeochemical cycles driven by microbes (11, 12). Further, rivers are among the most threatened ecosystems globally and face multiple human stressors related to land use and nutrient pollution (13–15). The concurrently enriched land-derived contaminants, along with sediment deposition, impact edaphic factors and microbial community and diversity in riverine islands, which in turn affect ecosystem stability and functions. Thus, uncovering ecological processes and drivers that shape the microbial communities is essential for understanding the ecosystem functions of riverine islands.

The Yangtze River, the longest river in Asia, runs through a 1.8 million-km^2^ watershed area and is home to 459 million people (16). Due to natural heterogeneous conditions at spatial and temporal scales in large river ecosystems, microbial communities are expected to display strong spatial patterns (17, 18). Moreover, at present, pollution discharge from agricultural land and land use change brought by urbanization are the main pressures faced by the middle and the lower Yangtze River (16, 19). As the environmental factors, such as levels of nutrients and organic matter, fluctuate with large fluvial discharge accordingly (20–22), the corresponding changes are mirrored by the composition and diversity of bacterial communities (23, 24). Thus, a question occurs as to how environmental factors influence the relative contribution of ecological processes to community assembly.

Here, we aimed to explore the patterns and drivers of microbial community assembly in riverine islands in the middle–lower Yangtze River. We addressed three main questions as follows: (i) the species composition and diversity of bacterial communities; (ii) the relative contribution of community assembly processes in shaping bacterial communities; and (iii) how environmental factors mediate variations in bacterial community composition and, in turn, influence environmental function profiles. This study is the first attempt to explore the microbial diversity patterns and community assembly mechanisms in riverine islands in large rivers. Therefore, the results of our study set a foundation contribution to the mechanism understanding of bacterial communities in riverine islands subject to varying natural and anthropogenic impacts.

## MATERIALS AND METHODS

### Study area and soil sampling

**Fig 1:**
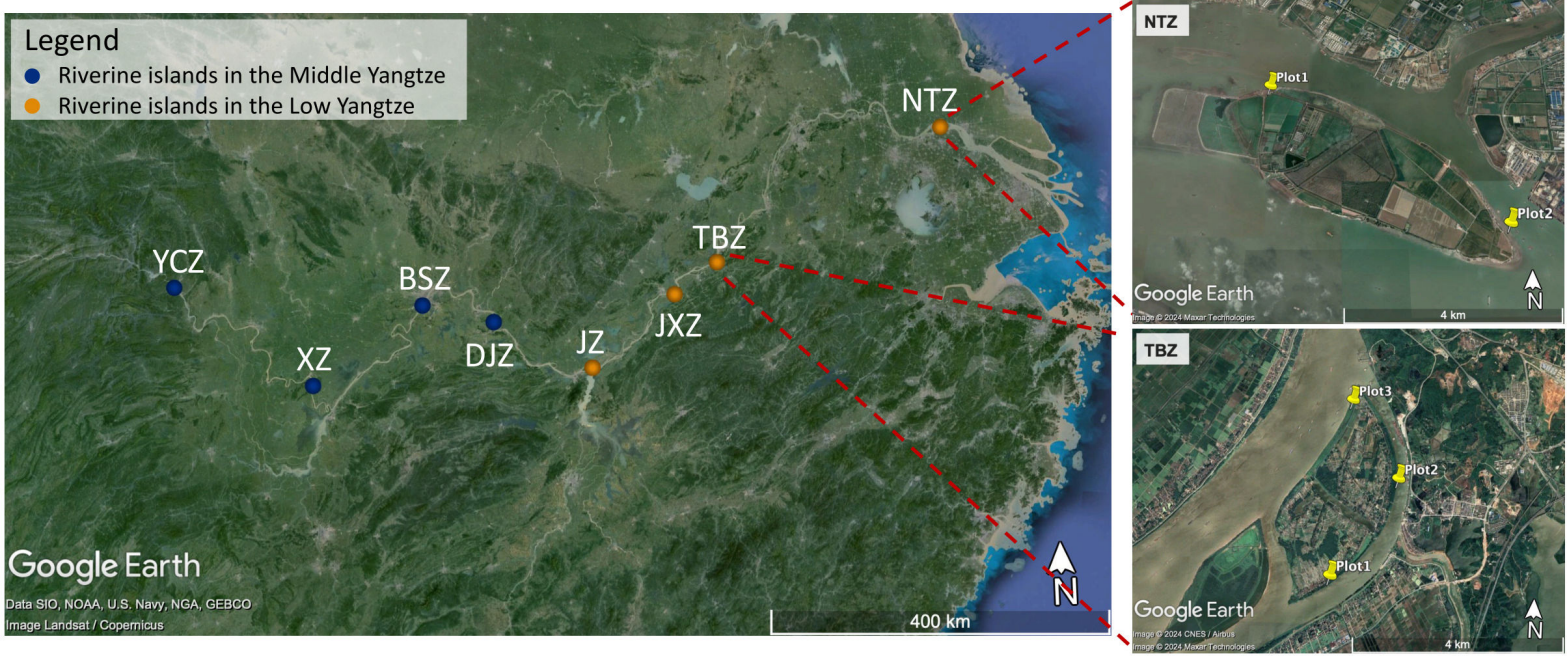
Locations of the eight study riverine islands in the middle–lower Yangtze River. Blue dots indicate riverine islands in the middle Yangtze (MR islands), yellow dots indicate riverine islands in the lower Yangtze (LR islands). YCZ, Yichangzhou island, three replicates; XZ, Xinzhou island, four replicates; BSZ, Baishazhou island, three replicates; DJZ, Daijiazhou island, four replicates; JZ, Jiangzhou island, three replicates; JXZ, Jiangxinzhou island, four replicates; TBZ, Tiebanzhou island, three replicates; NTZ, Nantongzhou island, two replicates (limited access due to rapid flow condition). Distribution of sampled sites were generated from Google Earth 7.3 using site geographic location (latitude and altitude).

Eight riverine islands were selected by random selection but also took into consideration their access (Table S1). One sampling campaign was conducted from 17 to 30 October 2021. Four study sites were in the middle of the Yangtze River, namely, Yichangzhou (YCZ, located in Yichang City), Xinzhou (XZ, located in Jianli City), Baishazhou (BSZ, located in Wuhan City), and Daijiazhou (DJZ, located in Huanggang City). The other four riverine islands were in the lower Yangtze River, namely, Jiangzhou (JZ, located in Jiujiang City), Jiangxinzhou (JXZ, located in Anqing City), Tiebanzhou (TBZ, located in Tongling City), and Nantongzhou (NTZ, located in Nantong City) (Fig. 1). The YCZ and NTZ were not officially named and thereafter were named according to their position. Distribution of sampled sites (Fig. 1) were generated from Google Earth 7.3 (the free version) using site geographic location (latitude and altitude). The mean annual temperature (MAT) and mean annual precipitation (MAP) observations were generated for each site from the WorldClim data product at a 30-s (~1 km^2^) spatial resolution (25). The MAT varied between 15.38°C and 17.31°C with a mean value of 16.85°C. The MAP ranged from 993 to 1486 mm with an average of 1,291 mm. We further obtained the mean temperature and mean precipitation in October (MOT and MOP) for each site from the Third Pole Environment Data Center (26, 27). The MOT and MOP varied from 18.0°C to 19.9°C (average 19.1°C) and 46.4 to 100.6 mm (averaged 65.28 mm), respectively.

In each riverine island, three or four replicate plots (5 × 5 m) were selected based on island area and shape, except for the NTZ (two replicates) due to its rapid flow condition and difficulty of access (Table S1). In each plot, five top-soil samples (0- to 10-cm depth directly below the litter layer) were taken randomly and homogenized to form a composite sample. After roots and residues were removed, approximately 500 g of sample was sealed and stored at 4°C in a refrigerator, while about 10 g of soil was frozen (–20°C) until DNA extraction within 1 week.

Analytical methods for soil chemical and physical properties have been described in detail by Yao et al. (28). Soil bulk density and moisture were measured by weighing fresh soil with a known volume ring, oven drying at 105°C for 24 h, and reweighing. Soil electrical conductivity (EC) and pH were measured in a 1:5 (soil:water ratio) mixture using a glass electrode. Soil inorganic nitrogen (${\text{NH}}_{\text{4}}^{\text{+}}\text{–N}$, ${\text{NO}}_{\text{3}}^{\text{–}}\text{–N}$, and ${\text{NO}}_{\text{2}}^{\text{–}}\text{–N}$) of field-moist soils was extracted with 2 M KCl and determined with a continuous flow auto-analyzer (EasyChem Plus, Systea, Italy). Soil organic matter of air-dried soil was determined by the loss-on-ignition method. Contents of soil total organic carbon and total nitrogen were quantified using a C/N analyzer (Elementar Vario MaxCube, Hanau, Germany). Soil total phosphorus was analyzed using the colorimetric method on a spectrophotometer (PerkinElmer, Waltham, USA) following a modified microwave-digestion method based on 3051A of the USEPA (1988) described in detail by Yao et al. (28). Soil texture was measured using a laser diffraction particle size analyzer (BT-9300ST, Bettersize Instruments Ltd., China) and partitioned into clay (0–2 µm), fine silt (2–6.3 µm), medium silt (6.3–20 µm), coarse silt (20–63 µm), and sand (63–1000 µm) categories. All parameters are expressed on an oven-dry weight basis (29).

### DNA extraction and amplicon-based sequencing

Total microbial genomic DNA was extracted from riverine island soils using the E.Z.N.A. soil DNA Kit (Omega Bio-tek, Norcross, GA, U.S.) according to the manufacturer’s instructions. The quality and concentration of DNA were determined by 1.0% agarose gel electrophoresis and an ultraviolet spectrophotometer (NanoDrop 2000, Thermo Scientific Inc., USA). Universal primers 338F (5′-ACTCCTACGGGAGGCAGCAG-3′) and 806R (5′-GGACTACHVGGGTWTCTAAT-3′) were applied to amplify the bacterial 16S rRNA gene. Sequencing was performed on the Illumina MiSeq platform at the Majorbio Bio-Pharm Technology Co. Ltd. (Shanghai, China).

### Data processing

Paired-end reads were de-multiplexed using an in-house Perl script and then quality filtered by fastp (version 0.23.2) and merged by FLASH (version 1.2.11) with the following settings: (i) The 300-bp reads were truncated at any site receiving an average quality score of <20 over a 50-bp sliding window, and the truncated reads shorter than 50 bp were discarded. Reads containing ambiguous characters were also discarded. (ii) Only overlapping sequences longer than 10 bp were assembled according to their overlapped sequence. Thereafter, the optimized sequences were clustered into operational taxonomic units (OTUs) with a 97% similarity cutoff using UPARSE (version 7.1). The most abundant sequence of each OTU was used to represent it. Taxonomy classification for OTUs was performed using the Naïve Bayes classifier trained on SILVA 138.1 Ref NR 99 (29, 30).

Samples were rarefied to 16,817 reads (minimum sequence number) to calculate the diversity indices (Shannon, Simpson, Chao, Ace, Pilou’s evenness were, and Faith PD) using the “vegan” and “ape” packages. The Functional Annotation of Prokaryotic Taxa (FAPROTAX) was used for functional annotation prediction in the soil, and the output functional table used the default settings (31). The bacterial OTUs were compared with the database obtained by FAPROTAX (script version 1.1).

### Statistical analyses

The Shapiro–Wilk test was used for normality test, and Levene’s test was used for homogeneity of variance test. Differences between means of soil properties were tested using the Kruskal–Wallis test with the Benjamini–Hochberg’s FDR (false discovery rate) corrections via the agricolae package (32). The analysis was performed using the lme4 (33) and multcomp (34) packages in R. The correlation analyses were conducted using the psych package (35), and the diagram was implemented using the pheatmap package (36).

Community composition across samples was compared using nonmetric multidimensional scaling (NMDS) based on the Bray–Curtis distance matrices. The Bray–Curtis dissimilarity metric was qualified using the “distance” function in the ecodist package. Permutational multivariate analysis of variance (PERMANOVA) was computed with 9,999 permutations using the “adonis” function. Similarity percentages species contributions analysis (SIMPER) was used to assess species contributing primarily to the observed difference between the two groups. Distance–dissimilarity relationship patterns between bacterial community and geographic distance or environmental distance were determined using Spearman correlation analysis. The weighted β-nearest taxon index (βNTI) and Bray–Curtis-based Raup–Crick (RC_bray_) values were calculated via a null model methodology to discern the ecological processes that drive bacterial community assembly (3, 37). The βNTI was calculated by the difference between observed phylogenetic community turnover and the mean of the null deviations. The RC_bray_ value was measured by the deviation between the observed Bray–Curtis matric and the null distribution via the vegan package (38). Specifically, |βNTI| > 2 indicates the dominance of deterministic processes that are associated with homogeneous selection (βNTI < −2) and heterogeneous selection (βNTI > 2). The βNTI value ranging between −2 and 2 (−2 < βNTI < 2) indicates the dominance of stochastic processes that are associated with homogenizing dispersal (RC_bray_ < −0.95), undominated processes (−0.95 < RC_bray_ < 0.95), and dispersal limitation (RC_bray_ >0.95) (5).

The influence of soil properties, climatic factors, or geospatial factors on microbial communities was assessed by redundancy analysis (RDA) using the “rda” function from the vegan package, with the microbial community data undergoing Hellinger transformation, and environmental factor data were log-transformed (except pH) using the “decostand” function from vegan. The significant explanatory variables were chosen by backward selection using “ordistep” and further manual deselection of variables if the variance inflation factor (VIF) is larger than 10 to avoid colinearity between environmental factors. The function “envfit” was run with 9,999 permutations to select the significantly related variables (*P* < 0.05). The VPA was further used to explore the influences of geospatial factors, climatic factors, and physicochemical factors on bacterial community composition. Prior to VPA, multicolinearity between environmental variables was performed using the VIF by the function “vif.cca” in the vegan package. Only variables with VIF <10 were included in further analyses.

Mantel tests were used to examine Spearman’s rank correlation between the monitored environmental factors and the bacterial community similarity using Bray–Curtis distance matrices with 9,999 permutations using the vegan package. Further, to assess the relationship between community composition and environmental factors or geographic distance after controlling for geographic distance or environmental factors, we performed a partial Mantel test with 9,999 permutations. To discern the relative importance of various environmental factors in structuring bacterial communities, a multiple regression on matrices (MRM) approach within the ecodist package was used. A full MRM including all environmental properties was calculated. Non-significant variables were removed stepwise from the full model.

## RESULTS

### Soil properties in different riverine islands

More than half of the measured and calculated environmental factors showed a significant difference between the middle Yangtze (MR) and lower Yangtze (LR) River islands (Table S2). In general, MAT, MOT, MOP, and C/N ratio were significantly higher in the MR islands than those in the LR islands. On the contrary, MAP, soil moisture content, contents of total nitrogen, total phosphorus, and ${\text{NO}}_{\text{2}}^{\text{–}}\text{–N}$ showed higher mean values in the LR islands. Furthermore, sediments from the MR islands had a coarser physical texture, with higher contents of sand as expected (Table S2).

### Spatial variation of bacterial community composition

**Fig 2:**
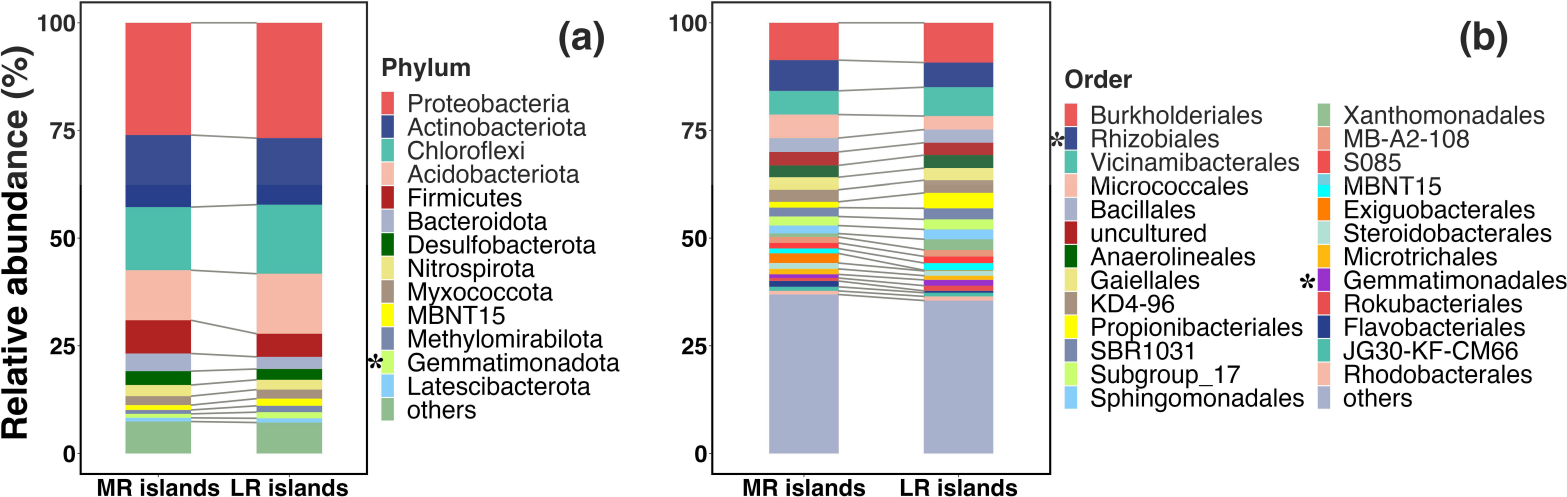
Relative abundances of bacterial community in riverine islands at (**a**) phyla and (**b**) order levels. Asterisk (_*_) indicate significant difference at *P* < 0.05 through the Kruskal–Wallis test.

Across all samples, a total of 8,729 bacterial OTUs were identified from 1,377,238 high-quality sequences at a 97% similarity level for the 26 samples. Overall, the most diverse OTUs in both MR and LR rivers were assigned to the groups of Proteobacteria, Actinobacteriota, and Chloroflexi (Fig. 2a). Specifically, the most common phyla of bacteria in the MR islands were Proteobacteria (average 26.1%), Actinobacteriota (16.7%), Chloroflexi (14.7%), Acidobacteriota (11.6%), Firmicutes (7.7%), Bacteroidota (4.1%), Desulfobacterota (3.2%), and Nitrospirota (2.6%), while the most dominated bacterial phylum in the LR islands was also Proteobacteria (26.8%), followed by Actinobacteriota (15.4%), Chloroflexi (16.1%), Acidobacteriota (14.0%), Firmicutes (5.4%), Bacteroidota (2.8%), Desulfobacterota (2.5%), and Nitrospirota (2.3%). Among the 13 relative abundances of the dominant phyla (>0.1%), only Gemmatimonadota was significantly different between the MR and LR islands (Kruskal–Wallis test, *P* < 0.05). At the order level, the most common bacteria in the MR islands were Burkholderiales, Vicinamibacterales, Rhizobiales, Propionibacteriales, Micrococcales, Bacillales, and Anaerolineales, while the most common order of bacteria were Burkholderiales, Rhizobiales, Vicinamibacterales, Micrococcales, Bacillales, uncultured bacterium, and Gaiellales in the LR islands (Fig. 2b). Among them, only Rhizobiales and Gemmatimonadales differed significantly between the MR and LR islands (Kruskal–Wallis test, *P* < 0.05). Those results implied that most soil bacterial taxa were not coupled to locations of the riverine islands in the Yangtze River.

**Fig 3:**
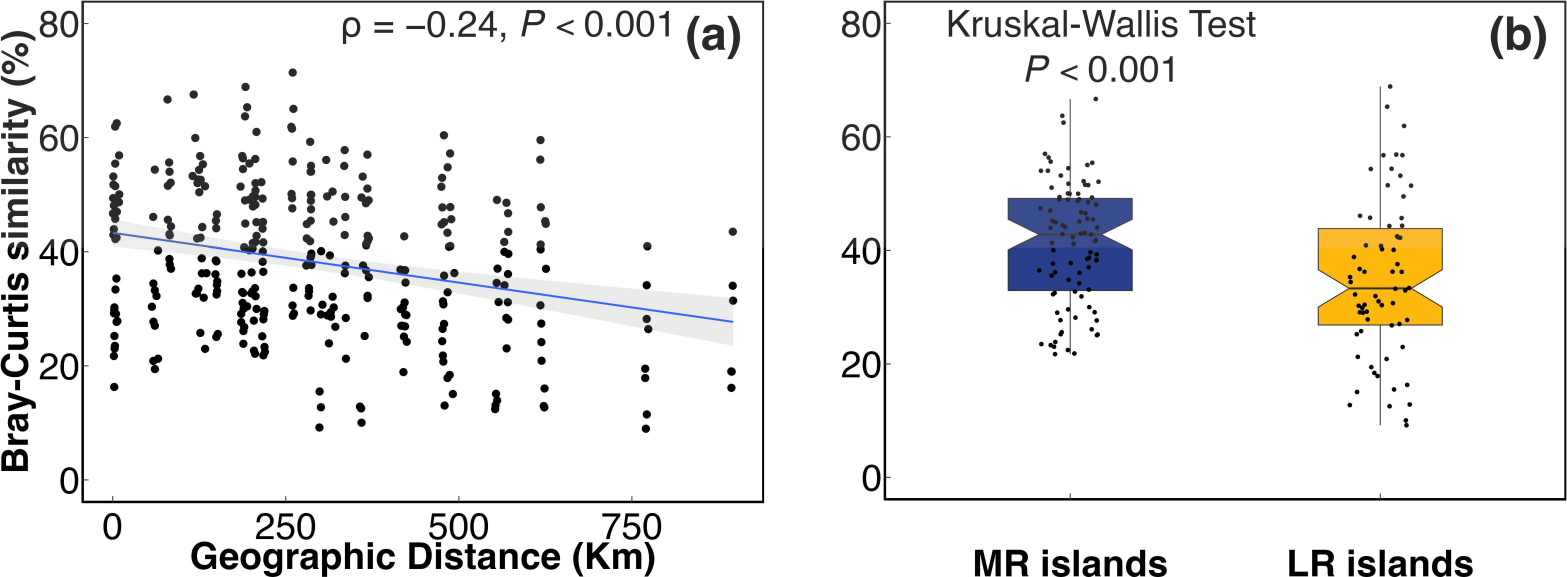
Distance–decay pattern and Bray–Curtis similarity of microbial communities in the riverine islands. (**a**) Distance–decay relationship based on Bray–Curtis dissimilarity along geographic distances for the bacterial community. (**b**) Comparison between Bray–Curtis similarity of the bacterial community in the MR islands and LR islands. *P* refers to Spearman’s rank correlations and statistical significances, respectively. Similarity was calculated based on 1-dissimilarity of the Bray–Curtis distance metric.

In the plot of NMDS ordination, sediment bacterial community composition varied according to their locations in the Yangtze River, whereas the separation could be only captured by a three-dimensional plot, especially on axes 1 and 3 (Fig. S1c), and the differences were not statistically significant (Fig. S1a, Adonis: *P* > 0.05; ANOSIM: *P* > 0.05). Compared to those in MR islands, significantly lower beta diversity values of the bacterial community were observed in the LR islands (Kruskal–Wallis test, *P* < 0.05, Fig. 3b; Fig. S1). The SIMPER analysis revealed that the top 10 OTUs contributing most to the difference in the community composition explained 10.79% of the difference (Table 1). Among them, only two OTUs differed significantly between the MR and LR islands (Table 1, Kruskal–Wallis test, *P* < 0.05), which were *Nocardioides* and *Lysobacter* at the genus level, while both were enriched in the LR islands than in the MR islands. Meanwhile, soil in the MR islands harbored bacterial community with higher richness, ACE, Chao1, and Shannon indices, but lower Faith’s PD than those in the LR islands, although the differences among the sampled riverine islands could not be statistically tested due to insufficient number of samples (Table S3).

**TABLE 1:** The SIMPER analysis showing the top contributing OTUs to Bray–Curtis distances of soil bacterial communities between MR and LR islands^*a*^

OTU code	Phylum	Order	Family	Genus	Average abundance (%)		P	The contribution of OTUs (%)	
					MR islands	LR islands		Contribution	Cumulative
OTU1	Actinobacteriota	Micrococcales	Micrococcaceae	Unclassified	0.33	0.15	0.517	3.10	3.10
OTU2	Firmicutes	Exiguobacterales	Exiguobacteraceae	*Firmicutes*	0.16	0.01	0.848	1.77	4.87
OTU3	Actinobacteriota	Propionibacteriales	Nocardioidaceae	Unclassified	0.03	0.12	0.060	1.12	5.99
**OTU4748**	**Actinobacteriota**	**Propionibacteriales**	**Nocardioidaceae**	***Nocardioides***	**0.02**	**0.11**	**0.039**	**0.95**	6.94
OTU16	Proteobacteria	Steroidobacterales	Steroidobacteraceae	Unclassified	0.06	0.06	0.652	0.69	7.63
OTU26	Acidobacteriota	Vicinamibacterales	Unclassified	Unclassified	0.05	0.06	0.533	0.66	8.29
OTU12	Firmicutes	Bacillales	Bacillaceae	*Bacillus*	0.04	0.08	0.109	0.65	8.94
**OTU6**	**Proteobacteria**	**Xanthomonadales**	**Xanthomonadaceae**	***Lysobacter***	**0.01**	**0.07**	**0.007**	**0.64**	9.58
OTU24	Proteobacteria	Sphingomonadales	Sphingomonadaceae	*Sphingomonas*	0.04	0.08	0.134	0.63	10.22
OTU18	Chloroflexi	KD4-96	KD4-96	KD4-96	0.06	0.07	0.447	0.57	10.79

^*a*^ *P* values are the significance from the Wilcoxon signed-rank tests. Values in bold indicate very significant differences in the relative abundance of the OTUs between MR and LR islands. MR islands: riverine islands in the middle Yangtze River; LR islands: riverine islands in the lower Yangtze River.

**Fig 4:**
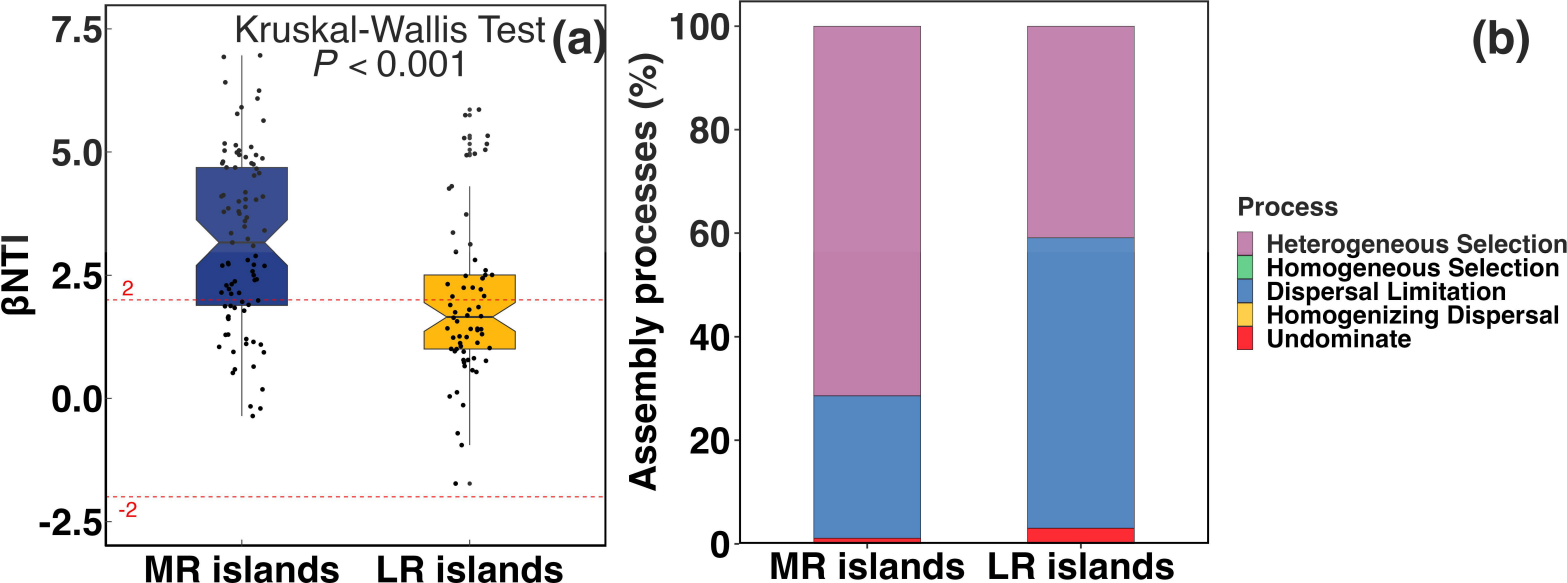
The bacterial community assembly processes in the riverine islands. (**a**) Comparison between the βNTI for sediment bacterial communities in the MR islands and LR islands. (**b**) Percentage of turnover in sediment bacterial community assembly.

In general, the sediment bacterial community decreased in similarity with increasing spatial distance (ρ = −0.24, *P* < 0.001, Fig. 3a). Significant differences in Bray–Curtis similarity of bacterial communities were observed between the MR and LR islands (Kruskal–Wallis test, *P* < 0.001, Fig. 3b). Compared to those in the MR islands, a significantly lower beta diversity value of bacterial community was observed in the LR islands. In addition, the similarity in bacterial community compositions in the MR and LR islands were related to different environmental factors (Fig. S3), with the former significance correlating with contents of ${\text{NH}}_{\text{4}}^{\text{+}}\text{–N}$, ${\text{NO}}_{\text{3}}^{\text{–}}\text{–N}$, ${\text{NO}}_{\text{2}}^{\text{–}}\text{–N}$, and total carbon content, while those in the LR islands were mainly related to pH, EC, and total carbon content (Fig. S3). The βNTI distributions gradually shifted from the MR islands to the LR islands, from deterministic process (βNTI > 2) in the former to stochastic process (−2< βNTI < 2) in the latter (Kruskal–Wallis test, *P* < 0.001, Fig. 4a). The relative contributions of each assembly process were quantified, and results showed that heterogeneous selection (71.4%) was dominant in the MR islands and then shifted toward mainly of dispersal limitation (56.1%) in the LR islands (Fig. 4b).

### Linkages of bacterial community composition with environmental factors

Constrained axes of the RDA on bacterial community composition were able to explain 24.8% of their variation. The first constrained axis of the ordination explained 14.0% of the total variation, while associated primarily with soil pH, moisture content, content of ${\text{NO}}_{\text{2}}^{\text{–}}\text{–N}\text{,}$ and MOP. The second axis reflected altitude and the content of ${\text{NH}}_{\text{4}}^{\text{+}}\text{–N}$ in soil, with an explanation of 10.8% (Fig. S2). Similarly, results of PERMANOVA revealed a significant proportion of the variation in bacterial community composition, which could be explained by soil moisture content, pH, concentration of ${\text{NO}}_{\text{2}}^{\text{–}}\text{–N}$, and MAT, with 9.4%, 7.8%, 7.6%, and 7.2%, respectively (Table S4).

The Mantel tests further demonstrated that the importance of environmental variables varied between the MR and LR islands. Particularly, in the MR islands, bacterial community diversity characteristics were significantly influenced by latitude, MOP, and contents of inorganic nitrogen (*P* < 0.05, Table S5), but the effect of latitude on bacterial community present a strong dependence on contents of inorganic nitrogen (*P* > 0.05, Table 2). By contrast, bacterial community in the LR islands were closely associated with pH, EC, contents of total carbon, and ${\text{NH}}_{\text{4}}^{\text{+}}\text{–N}$ (*P* < 0.05, Table S5). The influences of pH and EC on bacterial community in the LR islands were slightly stronger, when contents of total carbon and ${\text{NH}}_{\text{4}}^{\text{+}}\text{–N}$ (*P* = 0.001) were controlled, rather than the other way around in the partial Mantel tests (*P* = 0.001). The VPA results showed that the measured environmental factors explained a higher proportion of bacterial community variation in the LR islands (52.2%) than those in the MR islands (16.1%) (Fig. S3), while excluding environmental variables with VIF larger than 10. Specifically, in the MR islands, the soil edaphic factors and geospatial factors explained 14.8% and 2.0% of the community variations, respectively. For the LR islands, soil edaphic factors, climatic factors, and the shared effect accounted for 49.6%, 2.4%, and 0.2% of the community variations, respectively (Fig. S4). After the stepwise removal of non-significant variables in the MRM analysis, we found that the contents of ${\text{NH}}_{\text{4}}^{\text{+}}\text{–N}$ and ${\text{NO}}_{\text{3}}^{\text{–}}\text{–N}$ were significant factors influencing bacterial community composition in the MR islands, which explained 29.0% of the variation (*P* = 0.014), whereas pH and EC jointly explained 48.9% (*P* < 0.01) of the bacterial community composition in the LR islands (Table 3; Table S6). Overall, those results indicate that sediment chemical factors controlled the bacterial community composition in riverine islands, but the dominant influential factors were specific to sites.

**TABLE 2:** Partial Mantel test results showing the relationship between community composition and significant related environmental factors selected using the Mantel test

	Effect of selected variable on community composition	Controlling for	*R*	*P*
MR islands	Inorganic nitrogen	Latitude, MOP	0.49	0.008
	Latitude, MOP	Inorganic nitrogen	0.11	0.158
LR islands	Soil pH, EC	Total organic carbon, $NH_{4}^{+}-N$	0.55	0.001
	Total organic carbon, $NH_{4}^{+}-N$	Soil pH, EC	0.54	0.012

**TABLE 3:** Results of multiple regression on MRM analysis after the stepwise removal of non-significant environmental factors

	Environmental factors	Coefficient	*P*
MR islands (*R*^2^ = 0.290, *P* = 0.014)	$NH_{4}^{+}-N$	0.021	0.053
	$NO_{3}^{–}-N$	0.045	0.042
LR islands (*R*^2^ = 0.489, *P* = 0.007)	pH	0.206	0.037
	EC	0.002	0.008

### Bacterial functional annotation and distribution among different riverine islands

**Fig 5:**
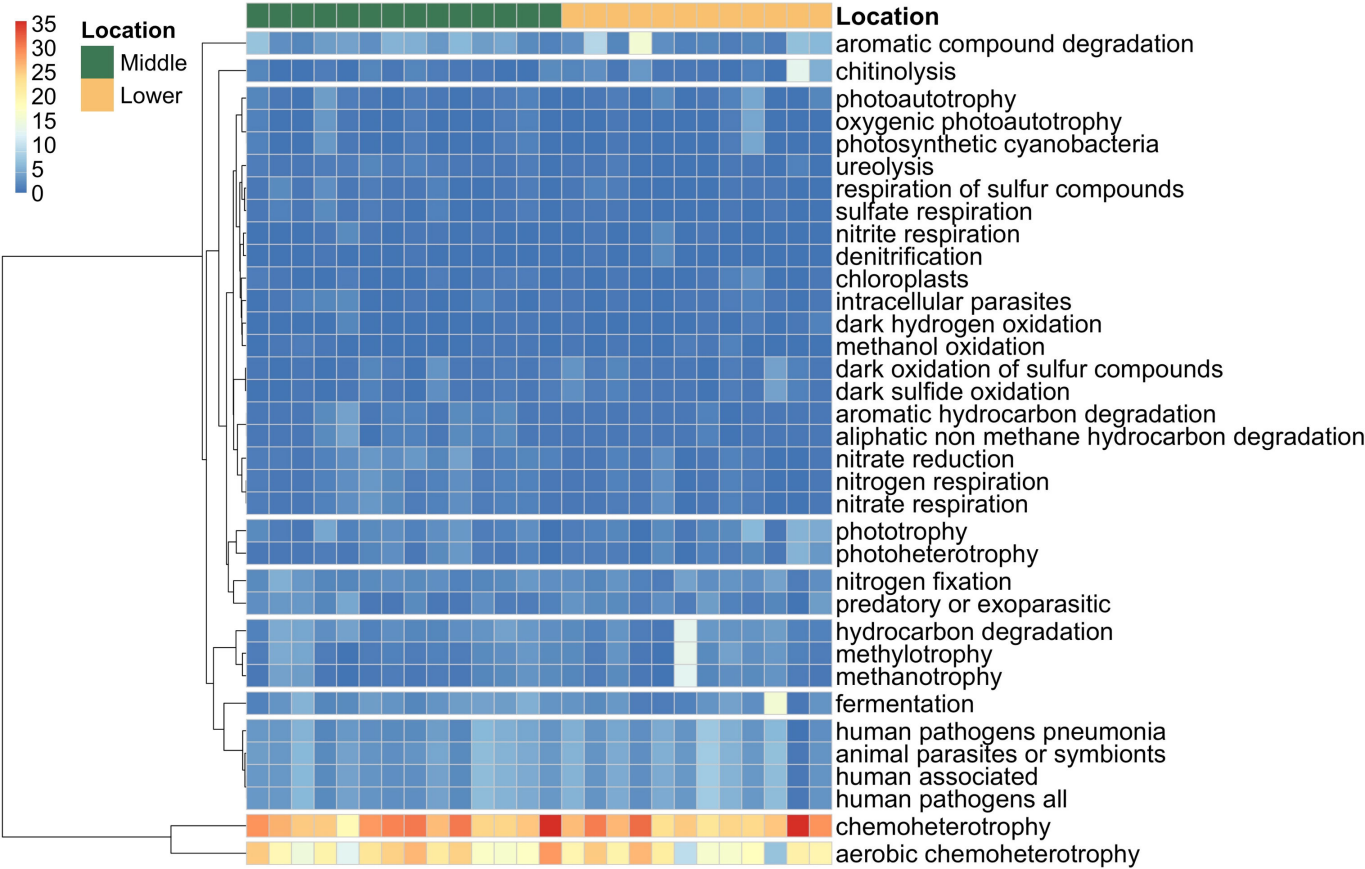
Distribution of the top 65 functional groups identified by the FAPROTAX database for different riverine islands in the middle–lower Yangtze River.

A total of 65 microbial functional groups were identified for bacteria in sediments from riverine islands in the Yangtze River according to the FAPROTAX database. The high-occurrence functional groups were visualized in Fig. 5. In general, aerobic chemoheterotrophs, chemoheterotrophs, and human-related pathogens prevailed in all eight sampled riverine islands. Notably, inorganic nitrogen respiration-related functional groups (nitrogen respiration, nitrate reduction, nitrate respiration, anammox) were significantly higher in the MR islands than those in the LR islands, but methane consumption-related functional groups (methanol oxidation, methylotrophy, methanotrophy) showed an opposite trend, although the differences were not statistically significant (Table S7).

## DISCUSSION

### Deterministic and stochastic processes jointly structured community assembly but their contributions varied

Distance–decay relationship reflects the landscape-scale species abundance distribution and can be used to compare and understand biodiversity patterns in diverse habitats (39). Variation of distance–decay slopes can be used to indicate the variability of spatial structure among habitats; a steep slope suggests a higher species turnover rate over space (40). The distance–decay relationship should be more feasible when dispersal is more limited, such as in soil systems. In the present study, the distance–decay relationship (ρ = –0.024, Fig. 3a) in the bacterial community composition was weak, with a turnover of 0.014 (fitness *R*^2^ <0.1). The fitness value of the relationship was substantially lower than the values reported in terrestrial ecosystems (–0.36 for bacteria in rice soils, –0.21 for bacteria in maize soils) (41–43), suggesting that the variation of spatial patterns of sediment bacterial community was less apparent in the ecosystem with high connectively. The possible reason was that the turbulent flow is more beneficial for the random movement of bacteria in river channels than in non-flowing habitats.

Assembly and succession of bacterial community are influenced by the interplay between deterministic and stochastic processes (3), but the relative importance of the two processes varied across habitats (44). In the present study, deterministic mechanisms strongly structured bacterial community assembly in the MR islands; over 70% of community turnover for bacteria was well explained by deterministic processes. In contrast, stochastic processes were responsible for nearly 60% of the community assembly in the LR islands. Increased stochastic processes in the LR islands indicate that the random movement of microbes, which progressively increased along the water flow, is the major driver of local community assembly. The above results are consistent with those of VPA, which showed that the edaphic factors explained much more variation in bacterial community compositions from the LR islands than those in the MR islands with proportions of 49.6% and 14.8%, respectively. Precipitation and soil moisture has been shown to influence the relative importance of deterministic and stochasticity assembly in bacterial community (45). Studies indicated that bacterial diversity tends to be higher in clay and silt fractions compared to the sand fraction (45, 46). This is attributed to the increased nutrient availability associated with fine particles, along with the protective environment of small pores that enhances conditions for bacterial colonization (9, 46). It is possible that the increased contents of clay and fine silt increased the available organic matter, total nitrogen, and phosphorus content, which in turn strengthened stochasticity processes in the LR islands.

### Community dissimilarity and importance of dispersal limitation increased concurrently along flow direction

Previous studies concluded that selection and dispersal limitation, other than homogeneous dispersal and drift, are the two major processes that influence patterns of spatial community dissimilarity (44, 47, 48). We found a higher beta diversity for sediment bacterial communities in the LR islands than those for the MR islands. Accordingly, heterogeneous selection dominated the community assembly processes in the MR islands (heterogeneous selection governing 71.4% and dispersal limitation governing 26.7%), whereas dispersal limitation was more important in the LR islands (heterogeneous selection governing 40.9% and dispersal limitation governing 56.1%). The observed community dissimilarity could be ascribed to the different importance of the four fundamental processes, especially environmental filtering and dispersal limitation. One possible explanation is the difference in the local environmental factors that imposed environmental filtering on bacterial communities (49). This is supported by our results that the measured environmental factors differed significantly between the MR and LR islands. The different contributions of heterogeneous selection in the MR and LR islands indicate that variance between environments introduced divergent selection pressures.

Further, in freshwater ecosystems, dispersal followed by species sorting has been reported to shape the composition of downstream river assemblages, and the balance of the two processes is associated with water flow and hydraulic conditions (50, 51). In this regard, another explanation could be the high volume of water flow and decent hydrodynamic conditions (in the lower Yangtze) ease the dispersal barriers and enhance the capacity of bacteria to colonize, thereby increasing the contribution of stochastic dispersal to bacterial assemblages in the LR islands. Previous studies also concluded that origins in upstream freshwater and terrestrial sources (seed banks) accounted for the biogeochemical patterns of bacterial communities in complex freshwater networks, including large rivers and lakes (51, 52). One more possible explanation could be elevated rare species that were driven by chance dispersal events, and their influence happened to be significant (53), which could be further supported by dispersal limitation primarily governing the bacterial community assembly in the LR islands, suggesting that the bacterial community was less limited by dispersal in the lower reaches of the Yangtze River. Overall, the bacterial community composition differed between the riverine islands in the middle and lower reaches of the Yangtze River, while the community dissimilarity and contribution of dispersal limitation to community assembly concurrently increased along the river flow direction.

### Distinct factors shape bacterial community compositions between the two riverine islands

An important ecological aspect for the understanding of biogeography is to determine the possible controlling factors and their contribution to the spatial variation of bacterial community. However, the role of local factors relative to regional factors often varies with spatial position in a river network (53). Specifically, bacterial community composition in the MR islands was found to be primarily related to the availability of inorganic nitrogen but less so to latitude and not significantly related to pH and EC, agreeing with the findings reported that structural changes of the bacterial community were significantly correlated with the available nitrogen in soil and sediment (54, 55). Strikingly, an insignificant association between sediment pH and bacterial community profiles was found in the MR islands. There are three likely reasons for this outcome. First, the clear influence of pH on the entire bacterial community composition might be offset by the counteracted association among pH and specific taxa, such as the significant negative relationship between pH and Actinobacteriota (second taxa in the top 13) but the positive relationships among pH and Nitrospirota, MBNT15, Gemmatimonadota, Latescibacterota, etc. Second, thresholds for the effect of pH on bacterial community might not be in the narrow pH (7.22–8.59) range of the four MR islands, since shifts in pH toward neutral conditions could lead to weaker environmental selection (6). Additionally, pH may indirectly alter bacterial community structure via interacting directly with other individual edaphic factors (e.g., nutrient availability, organic carbon characteristics, or salinity) (56, 57). Meanwhile, a large proportion of variation remains unexplained in the variation partition analysis, especially for those in the MR islands, although heterogeneous selection was the dominant process in bacterial community assembly. Thus, the unexplained portion could be attributed to drift, species interactions, and unmeasured environmental variables (2, 5). Large river ecosystems are extremely dynamic, and snapshot sampling generally introduces noise fraction, which may mask the main ecological patterns (58, 59). For example, Logares et al. (60) suggested environmental filtering (e.g., salinity) strongly drove bacterial community composition in lakes. Besides, Ruiz-González et al. (51) reported that hydraulic retention time determines the time that bacteria spend in a given ecosystem and therefore their capacity to grow in response to the local environment. In this context, the measured environmental factors in the present study were not fully responsible for the variation of bacterial community composition in the MR islands.

In contrast, pH and EC were the primary drivers of the variation in bacterial community composition in the LR islands. This general pattern is consistent with previous studies that soil pH is one of the strongest abiotic correlates of microbial communities (6, 7). Moreover, concentrations of sediment organic carbon and ${\text{NH}}_{\text{4}}^{\text{+}}\text{–N}$ were significantly associated with bacterial community composition, which is consistent with a previous study that reported that the demand for total carbon by bacterial community increased along the flow direction in the Hudson River ecosystem (22). In general, these results confirmed links between environmental constraints and bacterial community structures, although bacterial community composition was determined by distinct edaphic factors in the MR and LR islands.

### Assembly processes and human activities resulted in divergent bacterial community metabolism function

Similar to the change in community diversity, different microbial functional communities were formed in different riverine islands. We found significantly enriched metabolic functions associated with hydrogen degradation, nitrogen reduction, sulfur reduction, and iron respiration in the MR islands. The enriched nitrogen-related functional groups could be attributed to the dominant contribution of heterogeneous selection to bacterial assemblages in the MR island, while contents of inorganic nitrogen were the most important explanatory factors in community composition correspondingly. Similarly, previous studies showed that spatial differences in the functional structure of soil microbes could result from environmental selection for specific metabolic pathways based on physicochemical conditions (61, 62). Besides, both neutral and null model analyses showed that the phototrophic functional assemblages in soil are primarily controlled by stochastic processes (63). Accordingly, we found enriched phototrophic functional groups, while stochastic dispersal governed the bacterial community assembly processes in the LR islands. Thus, the assembly processes shaped the composition and diversity of bacteria and subsequently mirrored by biogeochemical cycles and metabolic function.

Sulfur cycling is generally linked with the biogeochemical cycles of carbon, nitrogen, and phosphorus, as well as the uptake and conversion of iron through iron respiration. Previous studies reported that aliphatic non-methane hydrocarbon degradation is associated with the sulfur cycles, and a major portion of overall sulfate reduction is likely driven by the consumption of non-methane hydrocarbons (64). Interestingly, we found that chlorate reducers were significantly enriched in the MR islands. Sodium chlorate has been used as a non-selective contact herbicide, defoliant agent, and even soil sterilant in agriculture (65). The capacity of chlorate reduction in river sediment, which had been exposed to higher than background concentrations of chlorate, is associated with the presence of chlorate-reducing bacteria (66). Indeed, in the Yangtze River catchment, agricultural land in the middle reach was 3.7 times larger than those in the lower reach (67).

On the other hand, the lower Yangtze River is characterized by an expanding economy and high demand for waterways. The Yangtze River Delta (YRD), located in the lower Yangtze River, is host to the busiest port cluster. Shipping emission inventory for the major ports in the YRD indicates that shipping is an important river pollution source, while oil residues, oily water, and sewage are produced during the operation of ships (68). Compared with the MR islands, the prevailing functional group of aromatic compound degradation in the LR islands was further corroborated by higher petroleum derivative pollution in the surrounding environments. We further identified the indicator taxa that were primarily responsible for the multivariate community patterns via SIMPER analysis. The results showed that *Nocardioides* and *Lysobacter* prevailed in the LR islands over the MR islands. Bacteria in the genus *Nocardioides* are capable of cure-oil degradation, steroid transformation, and industrial wastewater treatment (69, 70). In this regard, the increased number of *Nocardioides* may be partially explained by the transportation pollution source induced by inland waterway transport. Moreover, species in the genus *Lysobacter* have also been reported to reside in petroleum derivative-contaminated soil, heavy metals contaminated agricultural soils, inner-city river sediment, and freshwater lakes (70). In short, our results clearly suggested that some specifically critical metabolic functions were enriched and reflected the legacy effect of human land-use practice in the MR islands, whereas critical habitat-specificity bacterial taxa coping with human-related disturbances were enriched in the LR islands.

### Conclusion

This study revealed the ecological mechanisms underlying the bacterial community patterns and their functional profiles in riverine islands in the largest river in China. We detected differences in composition and functional diversity in the MR and LR islands, with substantial differentiation in deterministic and stochastic processes that jointly contribute to bacterial community assemblages. More importantly, we found that distinct environmental factors contributed to community composition, with inorganic nitrogen availability, in the MR islands, but pH and EC in the LR islands were the dominant explanatory factors. More generally, our study demonstrates that community assembly processes and local environmental factors can mediate community dissimilarity by selecting habitat-specificity taxa and particularly metabolism function and further highlights a mechanism by which species and functional groups may cope with intensified anthropogenic activities.

## Data Availability

The raw sequencing data have been deposited in the NCBI Short Read Archive database under accession number PRJNA1112345.

## SUPPLEMENTAL MATERIAL

Supplemental material spectrum.00818-24-s0001.docx Fig. S1 to S5; Tables S1 to S7.

ASM does not own the copyrights to Supplemental Material that may be linked to, or accessed through, an article. The authors have granted ASM a non-exclusive, world-wide license to publish the Supplemental Material files. Please contact the corresponding author directly for reuse.
