# Supplementary material for: Distinct drivers of bacterial community assembly processes in riverine islands in the middle and lower reaches of the Yangtze River
Source: Microbiol Spectr. 2024 Jun 13;12(8):e00818-24. doi: 10.1128/spectrum.00818-24 (PMC11302259; doi:10.1128/spectrum.00818-24)
Supplement: Supplemental material — Fig. S1 to S5; Tables S1 to S7. [file spectrum.00818-24-s0001.docx]

[**Figure S1** Nonmetric multidimensional scaling plots (NMDS) of bacterial composition in riverine islands in the middle reaches (MR islands) and riverine islands in the lower reaches (LR islands). 2](#_Toc156769634)

[**Figure S2** Results of redundancy analysis (RDA) showing relationships among the environmental variables. 3](#_Toc156769635)

[**Figure S3** Correlations between the Bray-Curtis similarity of microbial communities and environmental distance in riverine islands in the middle reaches (MR islands, a, b, c, d) and riverine islands in the lower reaches (LR islands, e, f, g, h, i, j, k). Figures are provided only when the spearman’s rank correlations were significant (*P* < 0.05). 𝜌, and P refer to spearman’s rank correlations and statistical significances, respectively. Similarity was calculated based on 1-dissililarity of the Bray-Curtis distance metric. 4](#_Toc156769636)

[**Figure S4** Variation partitioning analysis (VPA) showing the effects of geospatial, climatic and edaphic factors on sediment bacterial community in the MR islands (a) and LR islands (b). 5](#_Toc156769637)

[**Figure S5** Comparison between relative abundances of bacterial community in riverine islands at (a) phyla and (b) order levels. All data were presented as the mean ± SE. Asterisk (*) indicate significantly difference at P < 0.05 by Kruskal-Wallis test. 6](#_Toc156769638)

[**Table S1** Terrain information about sampling sites. A total of 8 riverine islands in the middle-lower Yangtze River were selected for sampling in October and November in 2021. 5](#_Toc145508967)

[**Table S2** Differences in environmental factors 6](#_Toc145508968)

[**Table S3** Alpha diversity of bacterial communities in soils collected from the eight riverine islands (no significant difference). 7](#_Toc145508969)

[**Table S 4** Results of permutational analysis to evaluate the impact of environmental factors on the bacterial community composition. 8](#_Toc145508970)

[**Table S5** Mantel tests for the correlations between environmental distance, individual environmental factors and bacterial community composition in riverine islands using Spearman’s coefficients. 9](#_Toc145508971)

[**Table S6** Results of the full multiple regression on matrices analysis (MRM) composed of all environmental factors. 10](#_Toc145508972)

[**Table S7** Differences in the abundance of predicted functional groups identified by the FAPROTAX database. 11](#_Toc145508973)

**Figure S1** Nonmetric multidimensional scaling plots (NMDS) of bacterial composition in riverine islands in the middle reaches (MR islands) and riverine islands in the lower reaches (LR islands).

**Figure S2** Results of redundancy analysis (RDA) showing relationships among the environmental variables. SOM = Soil organic matter, TOC = Total organic carbon, MOT = mean temperature in October, MOP = mean precipitation in October.


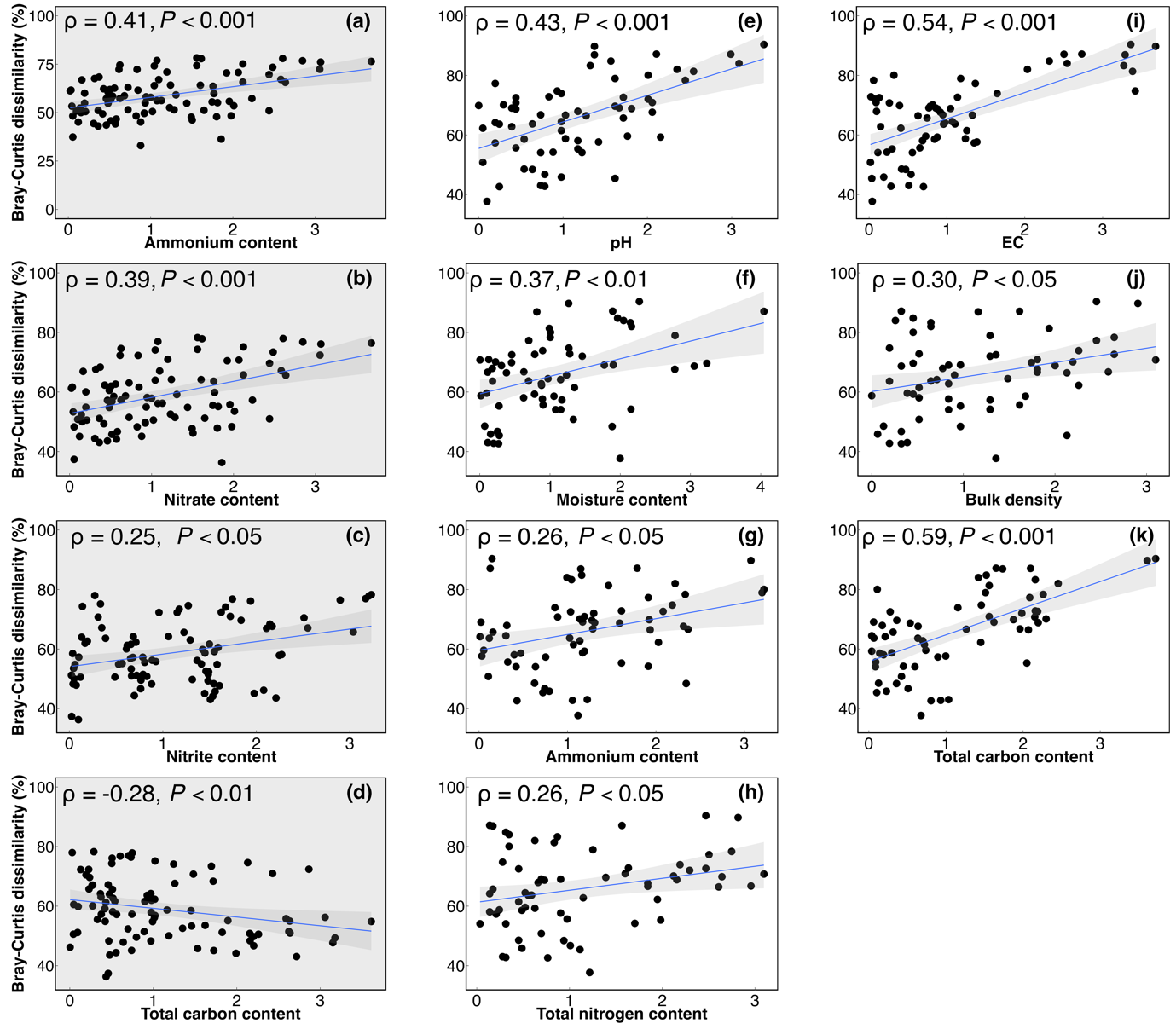


**Figure S3** Correlations between the Bray-Curtis similarity of microbial communities and environmental distance in riverine islands in the middle reaches (MR islands, a, b, c, d) and riverine islands in the lower reaches (LR islands, e, f, g, h, i, j, k). Figures are provided only when the spearman’s rank correlations were significant (*P* < 0.05). 𝜌, and P refer to spearman’s rank correlations and statistical significances, respectively. Similarity was calculated based on 1-dissililarity of the Bray-Curtis distance metric.


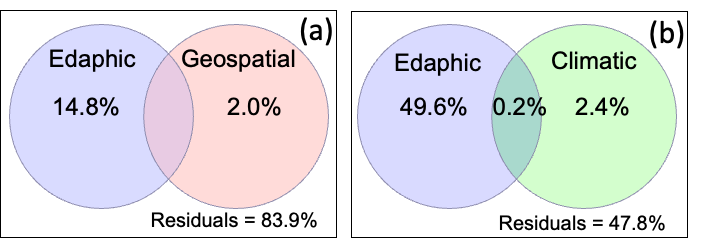


**Figure S4** Variation partitioning analysis (VPA) showing the effects of geospatial, climatic and edaphic factors on sediment bacterial community in the MR islands (a) and LR islands (b).


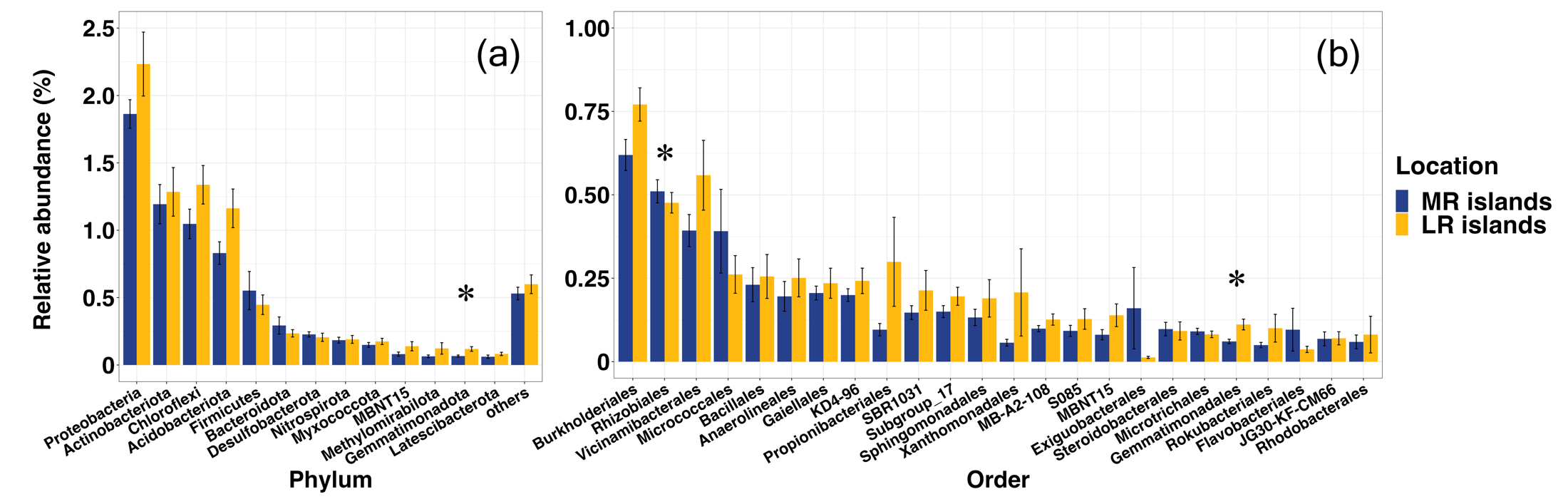


**Figure S5** Comparison between relative abundances of bacterial community in riverine islands at (a) phyla and (b) order levels. All data were presented as the mean ± SE. Asterisk (*) indicate significantly difference at *P* < 0.05 by Kruskal-Wallis test.

**Table S1** Terrain information about sampling sites. A total of 8 riverine islands in the middle-lower Yangtze River were selected for sampling in October 2021. MAT = mean annual temperature, MAP = mean annual precipitation, MOT = mean temperature in October, MOP = mean precipitation in October.

| Sites | Replicates | Longitude | Latitude | Altitude  (m) | MAT  (°C) | MAP  (mm) | MOT  (°C) | MOP  (mm) |
| --- | --- | --- | --- | --- | --- | --- | --- | --- |
| YCZ | 1 | 111.27 | 30.71 | 46 | 17.22 | 1085 | 19.1 | 46.5 |
| YCZ | 2 | 111.27 | 30.71 | 46 | 17.22 | 1098 | 19.1 | 46.5 |
| YCZ | 3 | 111.26 | 30.70 | 44 | 17.16 | 1093 | 19.1 | 46.4 |
| XZ | 1 | 112.89 | 29.80 | 24 | 17.12 | 1192 | 18.8 | 55.1 |
| XZ | 2 | 112.91 | 29.78 | 33 | 17.03 | 1203 | 18.8 | 55.3 |
| XZ | 3 | 112.93 | 29.77 | 25 | 16.97 | 1211 | 18.8 | 56.0 |
| XZ | 4 | 112.92 | 29.80 | 43 | 17.07 | 1202 | 18.8 | 55.8 |
| BSZ | 1 | 114.24 | 30.48 | 16 | 17.02 | 1240 | 18.1 | 47.3 |
| BSZ | 2 | 114.24 | 30.49 | 16 | 17.08 | 1247 | 18.1 | 47.3 |
| BSZ | 3 | 114.25 | 30.49 | 28 | 17.10 | 1248 | 18.1 | 47.4 |
| DJZ | 1 | 115.06 | 30.38 | 20 | 17.00 | 1339 | 19.2 | 50.4 |
| DJZ | 2 | 115.08 | 30.35 | 20 | 17.05 | 1355 | 19.3 | 50.9 |
| DJZ | 3 | 115.07 | 30.30 | 15 | 17.10 | 1358 | 19.2 | 49.8 |
| DJZ | 4 | 115.09 | 30.30 | 19 | 17.11 | 1355 | 19.3 | 50.0 |
| JZ | 1 | 116.22 | 29.77 | 11 | 17.30 | 1439 | 19.9 | 59.5 |
| JZ | 2 | 116.18 | 29.77 | 16 | 17.31 | 1426 | 19.9 | 59.1 |
| JZ | 3 | 116.12 | 29.77 | 13 | 17.30 | 1414 | 19.9 | 57.7 |
| JXZ | 1 | 117.17 | 30.50 | 25 | 16.65 | 1484 | 19.6 | 95.5 |
| JXZ | 2 | 117.20 | 30.53 | 15 | 16.61 | 1482 | 19.5 | 97.3 |
| JXZ | 3 | 117.22 | 30.53 | 12 | 16.60 | 1475 | 19.5 | 95.6 |
| JXZ | 4 | 117.22 | 30.50 | 13 | 16.66 | 1486 | 19.5 | 95.2 |
| TBZ | 1 | 117.72 | 30.80 | 6 | 16.50 | 1377 | 19.2 | 100.4 |
| TBZ | 2 | 117.73 | 30.83 | 11 | 16.51 | 1371 | 19.2 | 100.6 |
| TBZ | 3 | 117.74 | 30.82 | 4 | 16.52 | 1376 | 19.2 | 99.6 |
| NTZ | 1 | 120.54 | 32.03 | 5 | 15.39 | 993 | 18.4 | 64.5 |
| NTZ | 2 | 120.56 | 32.04 | 1 | 15.38 | 1027 | 18.4 | 67.5 |

**Table S2** Differences in environmental factors

|  | MR islands  (n = 14) | LR islands  (n = 12) |
| --- | --- | --- |
| MAP (mm) | **1230.43 ± 25.76^b^** | **1362.5 ± 49.16^a^** |
| MAT (℃) | **17.09 ± 0.02^a^** | **16.56 ± 0.18^b^** |
| MOP (mm) | **82.71 ± 5.44 ^a^** | **50.34 ± 1.00 ^b^** |
| MOT (℃) | **19.35 ± 0.15 ^a^** | **18.84 ± 0.12 ^b^** |
| pH | 8.12 ± 0.10 | 8.09 ± 0.06 |
| EC (us/cm) | **115.21 ± 9.69^a^** | **98.43 ± 9.96^b^** |
| Moisture content (%) | **30.18 ± 2.59^b^** | **35.73 ± 1.81^a^** |
| Bulk density (g/cm^3^) | 1.05 ± 0.05 | 1.15 ± 0.05 |
| SOM (mg/g) | 42.06 ± 3.46 | 45.48 ± 3.25 |
| Total organic carbon (mg/g) | 16.72 ± 0.74 | 16.32 ± 0.85 |
| Total nitrogen (mg/g) | **1.15 ± 0.06^b^** | **1.40 ± 0.08^a^** |
| Total phosphorus (mg/g) | **0.74 ± 0.07^b^** | **0.99 ± 0.02^a^** |
| $\text{NH}_{\text{4}}^{\text{+}}\text{–N}$ (μg/g) | 5.61 ± 0.53 | 5.04 ± 0.28 |
| $\text{NO}_{\text{3}}^{\text{–}}\text{–N}$ (μg/g) | 2.87 ± 0.24 | 2.87 ± 0.13 |
| $\text{NO}_{\text{2}}^{-}\text{–N}$ (μg/kg) | **27.29 ± 1.93^b^** | **35.29 ± 3.56^a^** |
| Clay (%) | **3.67 ± 0.43^b^** | **6.00 ± 0.52^a^** |
| Fine silt (%) | **9.75 ± 1.33^b^** | **15.53 ± 1.36^a^** |
| Medium silt (%) | **18.03 ± 2.38^b^** | **28.06 ± 2.47^a^** |
| Coarse silt (%) | **24.65 ± 2.37^b^** | **29.52 ± 1.03^a^** |
| Sand (%) | **43.89 ± 5.4^a^** | **20.88 ± 4.46^b^** |
| C:N ratio | **14.72 ± 0.57^a^** | **11.77 ± 0.41^b^** |
| C:P ratio | **28.37 ± 4.90^b^** | **16.59 ± 0.82^a^** |
| N:P ratio | 2.01 ± 0.39 | 1.42 ± 0.08 |

**Table S3** Alpha diversity of bacterial communities in soils collected from the eight riverine islands (no significant difference).

|  | Middle | Lower |
| --- | --- | --- |
| Richness | 2689 ± 111 | 2360 ± 178 |
| ACE | 4127 ± 198 | 3575 ± 296 |
| Chao1 | 4034 ± 188 | 3513 ± 289 |
| Shannon | 9.47 ± 0.18 | 9.29 ± 0.25 |
| Simpson | 0.99 ± 0.00 | 0.99 ± 0.00 |
| pielou | 0.83 ± 0.01 | 0.83 ± 0.01 |
| Faith’s PD | 155.2± 9.7 | 172.2 ± 5.4 |

**Table S 4** Results of permutational analysis to evaluate the impact of environmental factors on the bacterial community composition.

| Factor | R^2^ | F-value | *P* |
| --- | --- | --- | --- |
| Longitude | **6.77%** | **1.74** | **0.027** |
| Latitude | 5.13% | 1.3 | 0.180 |
| Altitude | 5.42% | 1.39 | 0.119 |
| Distance | **6.76%** | **1.74** | **0.030** |
| MAP | 5.75% | 1.45 | 0.083 |
| MAT | **7.24%** | **1.88** | **0.025** |
| MOP | 3.88% | 0.97 | 0.457 |
| MOT | 4.82% | 1.22 | 0.209 |
| pH | **7.78%** | **2.06** | **0.009** |
| EC | 5.81% | 1.48 | 0.076 |
| Moisture content | **9.37%** | **2.49** | **0.001** |
| Bulk density | 6.04% | 1.55 | 0.058 |
| SOM | 4.03% | 0.99 | 0.420 |
| Total organic carbon | 5.05% | 1.25 | 0.174 |
| Total nitrogen | 5.29% | 1.33 | 0.149 |
| Total phosphorus | 2.97% | 0.73 | 0.786 |
| $\text{NH}_{\text{4}}^{\text{+}}\text{–N}$ | 5.50% | 1.39 | 0.132 |
| $\text{NO}_{\text{3}}^{\text{–}}\text{–N}$ | 3.90% | 0.98 | 0.469 |
| $\text{NO}_{\text{2}}^{-}\text{–N}$ | **7.59%** | **1.99** | **0.009** |
| Clay | 5.25% | 1.32 | 0.126 |
| Fine silt | 4.69% | 1.18 | 0.224 |
| Medium silt | 5.56% | 1.42 | 0.095 |
| Coarse silt | 5.68% | 1.44 | 0.089 |
| Sand | 5.97% | 1.52 | 0.062 |
| C:N ratio | 5.90% | 1.51 | 0.071 |
| C:P ratio | 3.10% | 0.76 | 0.711 |
| N:P ratio | 2.93% | 0.72 | 0.746 |

**Table S5** Mantel tests for the correlations between environmental distance, individual environmental factors and bacterial community composition in riverine islands using Spearman’s coefficients.

|  | MR islands | | LR islands | |
| --- | --- | --- | --- | --- |
| Factor | r | *P* | r | *P* |
| Overall | -0.095 | 0.690 | **0.580** | **0.002** |
| Longitude | 0.029 | 0.403 | 0.238 | 0.131 |
| Latitude | **0.267** | **0.025** | 0.157 | 0.215 |
| Altitude | -0.014 | 0.511 | 0.142 | 0.261 |
| Distance | -0.004 | 0.437 | 0.256 | 0.136 |
| MAP | 0.004 | 0.450 | 0.376 | 0.074 |
| MAT | -0.194 | 0.879 | 0.164 | 0.209 |
| MOP | **0.229** | **0.048** | -0.127 | 0.892 |
| MOT | -0.030 | 0.469 | 0.201 | 0.160 |
| pH | -0.046 | 0.537 | **0.474** | **0.005** |
| EC | -0.155 | 0.804 | **0.665** | **0.002** |
| Moisture content | 0.087 | 0.283 | 0.361 | 0.100 |
| Bulk density | -0.055 | 0.625 | 0.318 | 0.058 |
| SOM | -0.215 | 0.916 | 0.137 | 0.189 |
| Total organic carbon | -0.251 | 0.960 | **0.620** | **0.004** |
| Total nitrogen | -0.123 | 0.705 | 0.281 | 0.111 |
| Total phosphorus | -0.154 | 0.801 | 0.025 | 0.401 |
| $\text{NH}_{\text{4}}^{\text{+}}\text{–N}$ | **0.446** | **0.031** | **0.335** | **0.043** |
| $\text{NO}_{\text{3}}^{\text{–}}\text{–N}$ | **0.430** | **0.019** | 0.089 | 0.300 |
| $\text{NO}_{\text{2}}^{-}\text{–N}$ | **0.332** | **0.041** | 0.149 | 0.160 |
| Clay | -0.316 | 0.986 | -0.051 | 0.652 |
| Fine silt | -0.340 | 0.991 | -0.101 | 0.831 |
| Medium silt | -0.296 | 0.986 | -0.085 | 0.749 |
| Coarse silt | 0.116 | 0.247 | 0.389 | 0.079 |
| Sand | -0.174 | 0.880 | -0.099 | 0.750 |
| C:N ratio | 0.207 | 0.085 | 0.297 | 0.068 |
| C:P ratio | -0.186 | 0.870 | **0.414** | **0.013** |
| N:P ratio | -0.145 | 0.761 | 0.190 | 0.170 |

**Table S6** Results of the full multiple regression on matrices analysis (MRM) composed of all environmental factors.

|  | MR islands  R^2^ = 0.52, *P* = 0.384 | | LR islands  R^2^ = 0.80, *P* = 0.003 | |
| --- | --- | --- | --- | --- |
| **Predictors** | **Coefficient** | ***P*** | **Coefficient** | ***P*** |
| Distance | 0.5178 | 0.750 | 0.4090 | 0.624 |
| MAT | -0.0005 | 0.768 | 0.0000 | 0.980 |
| MAP | 0.0001 | 0.932 | 0.0002 | 0.706 |
| MOT | -0.4923 | 0.123 | 0.1678 | 0.457 |
| MOP | 0.0063 | 0.117 | **-0.0024** | **0.010** |
| pH | 0.1650 | 0.642 | -0.2540 | 0.096 |
| EC | 0.0632 | 0.453 | 0.0608 | 0.427 |
| Moisture content | -0.0004 | 0.430 | **0.0020** | **0.005** |
| Bulk density | 0.0032 | 0.249 | 0.0074 | 0.094 |
| SOM | -0.0881 | 0.526 | -0.0345 | 0.901 |
| Total organic carbon | 0.0004 | 0.875 | -0.0028 | 0.352 |
| Total nitrogen | -0.0023 | 0.810 | 0.0058 | 0.633 |
| Total phosphorus | 0.0124 | 0.928 | 0.0429 | 0.789 |
| $\text{NH}_{\text{4}}^{\text{+}}\text{–N}$ | -0.0476 | 0.842 | **0.5099** | **0.022** |
| $\text{NO}_{\text{3}}^{\text{–}}\text{–N}$ | 0.0103 | 0.388 | 0.0233 | 0.344 |
| $\text{NO}_{\text{2}}^{-}\text{–N}$ | 0.0414 | 0.069 | 0.0094 | 0.869 |
| Clay | 0.0030 | 0.263 | 0.0012 | 0.470 |
| Fine silt | -0.0378 | 0.327 | 0.1111 | 0.178 |
| Medium silt | 0.0030 | 0.855 | -0.0426 | 0.221 |
| Coarse silt | -0.0032 | 0.720 | -0.0010 | 0.939 |
| Sand | 0.0000 | 0.994 | 0.0107 | 0.114 |
| C:N ratio | 0.0020 | 0.390 | 0.0010 | 0.877 |
| C:P ratio | -0.0008 | 0.945 | -0.0025 | 0.864 |
| N:P ratio | -0.0015 | 0.675 | -0.0133 | 0.187 |

**Table S7** Differences in the abundance of predicted functional groups identified by the FAPROTAX database.

| Functional groups | MR islands | LR islands |
| --- | --- | --- |
| chemoheterotrophy | 2723.07 ± 313.6 | 3168.42 ± 719.19 |
| aerobic.chemoheterotrophy | 2109.07 ± 276.13 | 2097.83 ± 438.43 |
| aromatic.compound.degradation | 351.07 ± 55.4 | 542.08 ± 221.67 |
| animal.parasites.or.symbionts | 325.07 ± 24.31 | 357.42 ± 44.66 |
| hydrocarbon.degradation | 241.64 ± 24.34 | 338.42 ± 146.36 |
| fermentation | 277 ± 28.91 | 254.5 ± 55.63 |
| chitinolysis | 87.14 ± 21.89 | 452.08 ± 309.41 |
| methylotrophy | 152 ± 22.55 | 318.33 ± 150.13 |
| phototrophy | 183.79 ± 41.71 | 276.75 ± 122.08 |
| nitrogen.fixation | 212.64 ± 17.83 | 223.08 ± 36.14 |
| methanotrophy | 121.79 ± 21.75 | 277.42 ± 142.26 |
| photoheterotrophy | 116.43 ± 29.7 | 210.33 ± 116.54 |
| nitrate.reduction | **179.79 ± 34.94a** | **72.67 ± 10.53b** |
| nitrogen.respiration | **136.86 ± 22.77a** | **63.83 ± 9.56b** |
| nitrate.respiration | **134.71 ± 22.75a** | **63.67 ± 9.52b** |
| dark.oxidation.of.sulfur.compounds | 75.21 ± 18.93 | 121.25 ± 30.43 |
| aromatic.hydrocarbon.degradation | **118.29 ± 25.95a** | **58.75 ± 9.45b** |
| aliphatic.non.methane.hydrocarbon.degradation | **117.64 ± 25.83a** | **58.67 ± 9.46b** |
| dark.sulfide.oxidation | 63.86 ± 18.01 | 113.25 ± 28.57 |
| photoautotrophy | 84 ± 30.16 | 89.25 ± 33.81 |
| ureolysis | 74.29 ± 10.6 | 60.83 ± 24.87 |
| oxygenic.photoautotrophy | 66.14 ± 28.86 | 54.83 ± 34.97 |
| photosynthetic.cyanobacteria | 66.14 ± 28.86 | 54.83 ± 34.97 |
| respiration.of.sulfur.compounds | **78.5 ± 19.73a** | **40.33 ± 7.41b** |
| sulfate.respiration | **66.29 ± 17.9a** | **28.5 ± 5.68b** |
| chloroplasts | 42.93 ± 8.04 | 50.17 ± 20.38 |
| dark.hydrogen.oxidation | 31.57 ± 8.11 | 46.5 ± 16.84 |
| nitrite.respiration | 39.71 ± 9.68 | 32.33 ± 8.29 |
| methanol.oxidation | 29.57 ± 3.89 | 40.92 ± 11.17 |
| denitrification | 23.86 ± 3.79 | 28.58 ± 7.76 |
| nitrate.denitrification | 23.86 ± 3.79 | 28.58 ± 7.76 |
| nitrite.denitrification | 23.86 ± 3.79 | 28.58 ± 7.76 |
| nitrous.oxide.denitrification | 23.86 ± 3.79 | 28.58 ± 7.76 |
| anoxygenic.photoautotrophy | 17.86 ± 2.41 | 34.42 ± 10.35 |
| anoxygenic.photoautotrophy.S.oxidizing | 17.86 ± 2.41 | 34.42 ± 10.35 |
| chlorate.reducers | **37.71 ± 12.55a** | **11.17 ± 5.5b** |
| dark.iron.oxidation | 28.21 ± 12.83 | 20.67 ± 7.69 |
| nonphotosynthetic.cyanobacteria | 21.71 ± 8.22 | 27.33 ± 5.39 |
| iron.respiration | **31.14 ± 8.78a** | **13.17 ± 4.83b** |
| aerobic.nitrite.oxidation | 14.21 ± 4.96 | 23.17 ± 6.29 |
| nitrification | 14.21 ± 4.96 | 23.17 ± 6.29 |
| sulfite.respiration | **20.14 ± 6.43a** | **6.25 ± 2.16b** |
| manganese.oxidation | 3.86 ± 0.95 | 24.25 ± 19.79 |
| sulfur.respiration | 8.57 ± 3.21 | 6.25 ± 5.08 |
| cellulolysis | 7.79 ± 1.53 | 6.58 ± 1.06 |
| fumarate.respiration | 9.57 ± 8.66 | 1.08 ± 0.48 |
| knallgas.bacteria | 9.57 ± 8.66 | 1.08 ± 0.48 |
| nitrate.ammonification | 9.57 ± 8.66 | 1.08 ± 0.48 |
| nitrite.ammonification | 9.57 ± 8.66 | 1.08 ± 0.48 |
| thiosulfate.respiration | 1.21 ± 0.37 | 3.33 ± 3.06 |
| xylanolysis | 2.36 ± 0.99 | 0.92 ± 0.36 |
| arsenate.detoxification | 1.57 ± 0.62 | 1 ± 0.35 |
| dissimilatory.arsenate.reduction | 1.57 ± 0.62 | 1 ± 0.35 |
| anammox | **2.14 ± 1.08a** | **0.17 ± 0.11b** |
| plastic.degradation | **1.14 ± 0.4a** | **0.25 ± 0.13b** |
| manganese.respiration | 0.43 ± 0.2 | 0.58 ± 0.26 |
| hydrogenotrophic.methanogenesis | 0.71 ± 0.57 | 0.08 ± 0.08 |
| methanogenesis | 0.71 ± 0.57 | 0.08 ± 0.08 |
